# Social Anxiety Modulates Subliminal Affective Priming

**DOI:** 10.1371/journal.pone.0037011

**Published:** 2012-05-17

**Authors:** Elizabeth S. Paul, Stuart A. J. Pope, John G. Fennell, Michael T. Mendl

**Affiliations:** 1 Centre for Behavioural Biology, School of Veterinary Science, University of Bristol, Bristol, United Kingdom; 2 School of Experimental Psychology, University of Bristol, Bristol, United Kingdom; University of Sydney, Australia

## Abstract

**Background:**

It is well established that there is anxiety-related variation between observers in the very earliest, pre-attentive stage of visual processing of images such as emotionally expressive faces, often leading to enhanced attention to threat in a variety of disorders and traits. Whether there is also variation in early-stage affective (i.e. valenced) responses resulting from such images, however, is not yet known. The present study used the subliminal affective priming paradigm to investigate whether people varying in trait social anxiety also differ in their affective responses to very briefly presented, emotionally expressive face images.

**Methodology/Principal Findings:**

Participants (n = 67) completed a subliminal affective priming task, in which briefly presented and smiling, neutral and angry faces were shown for 10 ms durations (below objective and subjective thresholds for visual discrimination), and immediately followed by a randomly selected Chinese character mask (2000 ms). Ratings of participants' liking for each Chinese character indicated the degree of valenced affective response made to the unseen emotive images. Participants' ratings of their liking for the Chinese characters were significantly influenced by the type of face image preceding them, with smiling faces generating more positive ratings than neutral and angry ones (F(2,128) = 3.107, p<0.05). Self-reported social anxiety was positively correlated with ratings of smiling relative to neutral-face primed characters (Pearson's r = .323, p<0.01). Individual variation in self-reported mood awareness was not associated with ratings.

**Conclusions:**

Trait social anxiety is associated with individual variation in affective responding, even in response to the earliest, pre-attentive stage of visual image processing. However, the fact that these priming effects are limited to smiling and not angry (i.e. threatening) images leads us to propose that the pre-attentive processes involved in generating the subliminal affective priming effect may be different from those that generate attentional biases in anxious individuals.

## Introduction

Damage to the primary visual cortex renders parts of the visual field inaccessible to consciousness (i.e. cortical blindness; [1]). Nevertheless, patients with these kinds of injuries are able to access some rudimentary information about images being viewed within their blind field, including the affective valence of primary visual reinforcers such as emotionally expressive faces [2]. This information is thought to be extracted via a secondary, sub-cortical visual pathway, which passes from the retina to the superior colliculus, pulvinar and extra striate visual cortex, by-passing the primary visual cortex [3] (but see also [4] for an alternative view). Similar processes also appear to function to extract affective information from emotive images in normally sighted humans, when paradigms are used which avoid primary visual cortex activation by showing images for extremely short durations (e.g. 10,20 or 30 ms), and backward masking them with additional images shown immediately afterwards [5]. It is now widely accepted that the evaluation of a visual stimulus can take place at a very early stage of processing (i.e. automatically, without conscious awareness – [6]), and such “subliminally” presented, masked emotive images have been found to have a number of effects on human observers, including the induction of autonomic arousal [7] and expressive facial musculature responses [8], and an accelerated capacity to make congruent evaluations of subsequent stimuli [9]. They have also been shown to cause rapid diversions of attention towards potential threats (e.g. to angry faces in a dot probe paradigms [10]), a process which appears to be modulated by the affective salience of the image to the observer. Specifically, a number of studies have found trait and clinical anxiety to be associated with rapid, pre-conscious orientation to potential threat images, linking anxiety to abnormally sensitive, early stage detection/evaluation of threat.

In a seminal paper in 1993, Murphy & Zajonc [11] showed that very briefly presented images of smiling and angry faces significantly influenced people's liking ratings for subsequently presented Chinese characters, and a number of researchers have since replicated this and similar “subliminal affective priming” (SAP) effects using a variety of emotionally expressive faces as primes (e.g. [12]–[15]). The SAP effect has been influential within psychological research because it demonstrates that unseen emotive face images can not only trigger peripheral facets of affective responding in the observer (as described above: facial expressions, alerting or vigilance responses, heightened disposition to make congruent evaluations), but that they can also generate a valenced affective response itself, in the form of an actual evaluation (positive or negative) of a subsequently appearing target. This is an important distinction. It shows that the rapid evaluations of visual stimuli made during early-stage, pre-attentive processing are made immediately available for affective judgements and decision making, in addition to serving as cues for attention orientation and action preparation [10]. Key questions regarding this process still remain to be fully investigated, however, including whether these resulting evaluations are binary or continuous (i.e. simply positive/negative, or varying levels of more/less positive and negative – [16]), whether or not the affective quality of these evaluations is actually consciously experienced by the observer (see [12] for discussion of this), and whether they vary between individuals according to the salience of the prime stimuli used (i.e. whether their effects are fixed and universal, or can be modulated by experience, trait dispositions, clinical disorders, etc.). The present study was designed to consider the last of these questions in particular: to establish whether the SAP effect varies significantly according to the trait-related salience of emotionally expressive face primes.

Facial expressions are regarded as primary reinforcers; they have intrinsic affective value to all human observers [17]. However, such stimuli are also more salient to some individuals than to others. In particular, people who suffer from clinically diagnosed social anxiety disorder (SAD; also known as social phobia) have been found to be especially likely to find facial expressions of emotion both impactful and attention-grabbing [18], [19]. SAD is characterized by a marked and persistent fear of social or performance situations in which the individual is exposed to unfamiliar people and/or the scrutiny of others [20]. A debilitating and relatively common psychiatric condition (lifetime prevalence rates in Western countries have been estimated to be as high as 12–13%, with proportionally more females that males reporting symptoms [21], [22]), sad can also be viewed as an extreme variant of a broader dimension of trait-based social anxiety in the wider population [23]. High levels of trait social anxiety, which can be assessed using a variety of self-report instruments, e.g. [24], [25], shares the key characteristics of SAD, including fear of social and performance situations, and anxiety about the scrutiny or evaluation of others [23].The present study was therefore designed to investigate whether the individual difference trait of social anxiety is associated with the extent of affective priming that occurs in response to subliminally presented, masked angry and smiling faces, the primes that have been most frequently and successfully used in SAP studies to date [11]–[13], [26]. Given that heightened concern over the negative scrutiny of others is a central feature of social anxiety, it is not surprising that both clinical and trait socially anxious observers have been found to differ from non-anxious ones in their responses to angry face images across a variety of paradigms (for recent reviews see [27], [28]). A number of studies have found heightened vigilance for angry faces in social anxiety using dot-probe tasks, in which briefly presented angry faces rapidly draw the visual attention of observers [29]–[31]. These findings also implicate trait social anxiety in variation in early-stage visual processing of facial expressions of anger in particular [30], and it has been shown that highly trait socially anxious individuals will fixate more rapidly than low socially anxious ones on both angry and happy faces, as compared to neutral faces [32]. What is still not known, however, is whether trait social anxiety is also associated with variation in the affective consequences of such early stage, pre-cortical processing. That is, we do not know whether these modulations of visual attention or vigilance are also accompanied by modulations of the valenced (positive or negative) effect these images have, as can be measured by their impact on immediately subsequent evaluative processing (e.g. influencing liking and disliking of stimuli within the SAP paradigm). We therefore designed the present study to investigate whether naturally occurring variation in social anxiety in a student population is associated with tendency to show subliminal affective priming to emotionally expressive faces (angry and smiling).

An additional issue concerns whether, and to what degree, the very brief affective responses derived in this manner are actually consciously experienced [15], [33]. If they are, they may be influenced by individual variation in a general tendency to attend to feelings, emotions, or affective states. In recent years, there has been a growing interest in such individual variation, assessed using self-report inventories such as the Mood Awareness Scale, a measure of the tendency to attend to and consider one's own affective states [34] (see also [35] for details of associations between mood awareness and other measures of emotional awareness). Because any affective responses that are measured in the SAP paradigm as liking judgements are likely to be both small in magnitude, and very short lived, trait differences between individuals in their capacity or tendency to consciously detect brief and/or subtle affective feelings (i.e. by diverting attention to them) may be particularly sensitive to such stimuli. Subliminal affective priming is thought to be mediated via visual images that are not consciously experienced, but whether the evaluations made as a result of witnessing these unseen primes are consciously experienced or not remains to be conclusively established. Murphy and Zajonc [11] originally proposed that the SAP effect was likely to be caused by fleeting affective feeling states misattributed to the Chinese character masks that followed the unseen facial primes (i.e. making use of “affect as information” [36]); if this is so, we would expect more mood aware individuals to show a greater SAP effect, because they have better chance of detecting these fleeting feeling states. If, in addition, socially anxious individuals also show greater mood awareness, we could find social anxiety to be linked to the SAP effect simply by virtue of the greater attention paid by such individuals to these briefly experienced affects. To explore this issue further, and to rule out the possibility of a confound between mood awareness and social anxiety, we measured trait mood awareness in addition to social anxiety, to investigate whether a tendency to focus on emotional feelings could enhance the subliminal affective priming effect.

Although a number of previous experiments have shown affective priming to occur in conditions where facial primes have been described as “unseen” or “subliminal”, none to date have experimentally confirmed that the emotive primes used were actually visually indiscriminable from their matched, neutral-face comparison primes (some previous studies have used forced-choice awareness tests of the identity, but not expression, of the faces viewed – e.g. [13], [14]). For the purposes of the present study, it was considered important that the affective primes used were not visually discriminable from one another, so that any associations demonstrated between the trait variables measured and affective priming effects could be interpreted as directly concerning a valenced affective response, and not one derived perceptually but non-affectively from visual registration of the expressive face stimuli themselves (e.g. giving rise to participant compliance or expectancy effects, or semantic congruency effects). By designing an affective priming task in which the discriminative stimuli (angry vs neutral and smiling vs neutral) were below both the subjective and objective thresholds for visual detection, we sought to ensure that any affective priming effects found were mediated via non-conscious visual processes [37], and that the emotional processing being targeted would thus be immune from any higher-level influences or expectations mediated via the conscious recognition of smiling or angry faces [38]. Exposure durations for the objective and subjective visual discrimination of masked smiling and neutral faces, and angry and neutral faces were therefore established in a pilot study prior to commencement of the main experiment. For our main experiment, we were then able to investigate whether trait social anxiety (and/or mood awareness) was associated with affective priming to genuinely “unseen” smiling and angry face images.

## Methods

### Pilot study: establishment of thresholds for discrimination of emotive and neutral face images

This initial study sought to establish visual detection thresholds for the smiling and angry faces, masked by the Chinese characters, which were to be used in the main subliminal affective priming (SAP) experiment to follow. A range of display durations (10 to 70 ms) were investigated, with participants' capacity to distinguish between emotive (smiling or angry) and neutral faces being assessed at each of these durations.

Fifteen volunteers (11 female; mean age 33.33 years, sd 8.07) participated in a computer-based task (for equipment information, see below) in which they were required to distinguish images of masked smiling and neutral faces using a two-alternative forced choice paradigm. A separate group of 15 participants (11 female, mean age 31.07 years, sd 8.04) distinguished masked angry and neutral faces. The masks were Chinese characters taken from PMingLiU font in Microsoft Word (point size 160), and the faces were from the Ekman and Friesen photographic picture set [39]. Participants were told that the emotive and neutral faces were “hidden” behind each Chinese character mask, and that while some would be clearly visible, others would be shown so briefly that they may not be seen at all. The experiment took place in a dimly lit room. Each trial consisted of two presentations of a prime face, followed by (the same, randomly chosen) Chinese character; one trial was always primed with a smiling/angry face expression and the other with a neutral expression from the same individual, order counterbalanced across trials. Each task consisted of 140 trials: each of 10 face pairs (5 male and 5 female) shown twice for each time duration, yielding a total of 20 trials per duration. For each of the two tasks (angry vs neutral; smiling vs neutral), ten individuals' face images were pictured with both an angry/smiling expression and a neutral expression, providing twenty identity-matched stimuli in total for each of 7 prime durations (10, 20, 30, 40, 50, 60, 70 ms). The Chinese character masks were shown for 2000 ms. Within-trial intervals between each of the two prime/mask presentations were 500 ms. Following each presentation pair, a question was displayed on the screen, asking participants to indicate (using the computer keyboard) which presentation contained the smiling/angry face, and how confident they were in this judgement (“sure”, “maybe”, “guess”).

Binomial tests were conducted to establish whether participants were able to distinguish between smiling/angry and neutral face primes at above-chance levels for each of the durations tested (10 to 70 ms). For both smile and neutral face, and angry and neutral face discriminations, the objective threshold for above chance levels of detection was between 10 ms and 20 ms (i.e. participants correctly distinguished the emotive face for 10 or more of the 20 trials per exposure duration, regardless of confidence level: Smile & neutral face 10 ms: 2 tailed p = 0.302; 20 ms: 2 tailed p<.001; Angry & neutral face 10 ms: 2 tailed p = 0.607; 20 ms: 2 tailed p<.05). Participants distinguishing between smiling and neutral face primes at 20 ms durations were significantly more often correct in their judgements than were those participants who were required to distinguish angry and neutral face primes at 20 ms (mann-Whitney u = 58.5, p<.05). For smile and neutral face discriminations, the subjective threshold for above chance levels of correct and “sure” detection was between 30 and 40 ms (30 ms: 2 tailed p = 1.00, 40 ms: 2 tailed p<.001). For angry and neutral face discriminations, a significant subjective threshold for above chance levels of correct and “sure” detection was not achieved, even at 70 ms prime durations (40, 50, 60, 70 ms: 2 tailed p = 0.118). It was concluded that for the main experiment to follow, the majority of observers would not be able to visually differentiate masked emotive (smiling, angry) and neutral expressions displayed for 10 ms duration, using the current apparatus and images.

### Main Experiment: Is Subliminal Affective Priming Modulated by Social Anxiety?

**Ethics statement:** Each participant provided informed written consent to take part in the experiment, and was fully debrief following the experiment. Data provided by participants were recorded and stored anonymously. The study was conducted in accordance with the principles expressed in the Declaration of Helsinki and was approved by the University of Bristol Faculty of Medical and Veterinary Sciences Committee for Research Ethics.

**Participants:** Eighty students and staff at the University of Bristol volunteered to undertake the experiment, of whom 67 fully completed the SAP task (see Result section for details). Requirements for participation were normal or corrected to normal vision and no knowledge of Chinese script. Participants were given a book token as thanks for completion of the task.

**Equipment:** The same computer hardware and software was used in the pilot study and main study: Viglen personal computer with Pentium 4 HT dual processors running at 3.4 GHz; 3.25 GB Memory; Iiyama vision master pro 454 HM903DT B 19 inch Diamondtron CRT monitor; ATI Radeon X700 SE video card; Windows XP Professional (SP2) and E-Prime psychological testing software (SP3) 1.1.4.4 [40]. The screen resolution used was 800×600 px. The monitor refresh rate was set at 200 Hz, providing a new screen refresh every 5 ms. The briefly presented face images were programmed to be pre-released by e-prime software to ensure minimal onset delays. A photo diode and oscilloscope were used to confirm exposure durations of all face image displays (10 ms duration).

**Stimuli and experimental design:** The computer task was designed to assess the effects of angry, smiling and neutral face primes on participants' liking judgements for subsequently presented Chinese character masks. The prime stimuli used were monochrome photographs of ten individuals' faces, five male and five female, from the Ekman and Friesen picture set [39]. For each of these photographed individuals, there was one angry, one smiling and one neutral exemplar (30 images in total). Each trial consisted sequentially of a 1000 ms fixation cross, 10 ms face prime, 2000 ms Chinese character mask, and a report screen on which participants were asked to rate their liking of the preceding Character on a scale of 1 (“didn't like it at all”) to 8 (“liked it a lot”). This screen terminated when a rating had been made, and no rating could be made prior to the appearance of the rating screen. The inter-trial interval was 2000 ms. Participants were exposed to the 30 prime-mask pairs in random order. For each face prime, a Chinese character mask was randomly selected (without replacement) from a pool of 60 Chinese characters, thereby ensuring that a broad range of rated mask stimuli were used, and that no characters were repeated for individual participants (see [12] regarding problems associated with repeated used of Chinese characters across primes). The 60 characters used had previously been selected from a larger pool of 120 (taken from PMingLiU font in Microsoft Word), on the basis of having been rated as most affectively neutral by a separate panel of 5 volunteers.

**Procedure:** Participants were tested individually in a dimly lit experimental room. Prior to undertaking the task, they completed two self-report scales: the Liebowitz Social Anxiety Scale [25] (LSAS; comprising two parts, in which participants rate their fear of (Part 1), and tendency to avoid (Part 2), the same list of social situations; see also [41] for further details of the psychometric properties of this scale) and the Mood Awareness Scale [34]. The images on the computer screen were 18 cm high, 14 cm wide, and viewed at an approximate distance of 45 cm (vertical and horizontal visual angles of 11.3° and 8.8° respectively). Instructions were provided on screen, informing participants that they were about to view a series of 30 Chinese characters, and that they would be required to rate their liking for each one on an eight-point scale. They were not told that face primes were being shown prior to the Chinese character masks. Instead, they were informed that the study was designed to investigate individual variation in liking for different types of image, and they were assured that there were no right or wrong answers – it was simply their own opinions that were being sought. Before starting the main task, participants were given a practice session in which they rated 4 neutral face primed Chinese characters. They then proceeded to the main experiment, in which they rated their liking for each of the 30 primed Chinese characters (10 neutral face primes, 10 smiling faced primes, 10 angry face primed). At the end of the task, participants were asked if they had noticed any images appearing on the screen aside from the characters they were rating. They were then fully debriefed and thanked for their participation.

## Results

Eight participants' data were removed from the study because of failure to complete the task, or failure to complete the trait questionnaires (MA and LSAS). A further 5 participants were excluded from the final data analyses because in the debriefing session they reported that they either thought they had seen faces during the task (n = 3) or guessed that faces preceded the Chinese character displays (n = 2). Two participants failed to complete Part 2 of the LSAS (avoidance scale), so their mean LSAS scores were based on their LSAS Part 1 (fear scale) score only (this was deemed acceptable as the two parts of the scale were well correlated (r = .46, p<.001), and had very similar means (24.4 and 25.5 respectively)). This left a total sample of 67 participants (47 female; mean age 34.12 years, sd 12.16).

Across the entire sample, the mean of liking ratings for the smiling face primed Chinese characters was 4.78 (s.d. = 0.81) and those for the angry and neutral face primed characters were 4.70 (s.d. = 0.93) and 4.70 (s.d. = 0.94) respectively. Mean response times for making liking judgements about angry, neutral and smiling face primed Chinese characters were 1470 ms (s.d. 601 ms), 1434 ms (s.d. 616 ms) and 1515 ms (s.d. 707 ms). The overall mean LSAS score was 25.10 (s.d. 8.15), with responses showing a close-to-normal distribution, but with a small positive skew (z = 2.177, p<.05). Mood awareness scores showed neither skew nor kurtosis, with a mean of 41.22 (s.d. 8.99); they were not correlated with LSAS scores (Pearson's r = −.024, p = .845).

A within-subjects GLM, with LSAS and MA scores as covariates, was used to investigate the effects of angry, smiling and neutral face primes on the participants' ratings of Chinese characters. A significant effect was found for prime type (F(2,128) = 3.107, p<.05, ω^2^ = .006), indicating a small affective priming effect, although post-hoc tests did not reveal any significant differences between character ratings preceded by angry vs. neutral, smiling vs neutral, nor angry vs smiling face primes (Bonferroni and LSD, all p>.25). Neither participants' mean LSAS scores, nor their MA scores, were significantly associated with liking ratings overall (F(1,64) = 0.994, p = .323; F(1,64) = 0.956, p = .332, respectively), but there was a significant interaction between LSAS scores and prime type (F(2,128) = 3.674, p<.05, ω^2^ = .008). There was no significant interaction between prime type and MA scores (F(2,128) = 1.141, p = .323).

To further investigate the relationship between social anxiety and affective priming, additional scores were generated for each participant for the relative impact of smiling as compared to neutral face primes on liking judgements (mean liking rating for smile-primed characters minus mean liking rating for neutral-primed characters; S-N liking), the relative impact of angry compared to neutral face primes (mean liking rating for angry primed characters minus mean liking for neutral-primed characters; A-N liking), and the relative impact of smiling as compared to angry face primes (mean liking rating for smile-primed minus mean liking rating for angry face primed characters; S-A liking). Pearson's correlations were then conducted to identify associations between these variables and participants' scores on the LSAS and MA scales. S-N likingwas positively correlated with LSAS (Pearson's r = .323, p<.01, n = 67), indicating that participants reporting higher levels of social anxiety rated smile-primed Chinese characters more positively than neutral-primed ones. S-A liking showed a near-significant positive correlation with participants' LSAS scores (Pearson's r = .237, p = .054, n = 67), but there was no association between A-N liking and LSAS scores (r = .069, p = .579, n = 67). MA was associated with neither S-N liking, A-N liking, nor S-A liking (Pearson's r = .166, p = .180; r = .014, p = .908; r = .143, p = .248; n = 67).

An additional GLM analysis was conducted to investigate the possible relationship between prime type, LSAS and MA scores, and participants' mean response times for making liking judgements about angry, neutral and smiling face primed Chinese characters. This revealed no significant effects for prime type, mean LSAS scores or MA scores (F(2,128) = 1.947, p = .147; F(1,64) = 0.486, p = .488; F(1,64) = 1.279, p = .262). There were no significant interactions between mean LSAS scores and prime type (F(2,128) = 0.085, p = .919), or MA and prime type (F(2,128) = 2.839, p = .062), in the determination of response times.

## Discussion

Our main experiment replicated the findings of previous research by showing a small but significant subliminal affective priming (SAP) effect, in which displays of Chinese characters were rated as more liked when they were immediately preceded by images of smiling (as opposed to angry or neutral) faces. We also demonstrated for the first time that this is achievable even when the emotive and control prime stimuli used have been shown to fall below subjective and objective visual discrimination thresholds. That is, we showed that subliminal, and not just sub-optimal primes [11], [13], are able to produce an affective priming effect. In accordance with our hypothesis, we found that the trait of social anxiety was associated with subliminal affective priming; specifically, higher self-reported levels of social anxiety in a non-clinical, student population were linked to higher preference ratings for Chinese characters following smiling face primes relative to those made following neutral face, control primes. The trait of mood awareness was not linked with self-reported social anxiety, and was not significantly associated with the modulation of liking decisions by emotive primes, indicating that self-reported individual variation in the tendency to focus on or attend to emotional/affective feelings does not play a part in moderating the affective impact of early-stage processing of emotive face images and does not represent a confound of trait social anxiety.

Our finding that smiles but not angry faces generated a priming effect was initially surprising, given that we had hypothesised that angry facial expressions would have most pre-attentive salience to socially anxious participants (i.e. as social threat stimuli), and would therefore be most likely to have their effects modulated by trait social anxiety. It is consistent, however, with a number of previous studies that have found SAP based on smiling faces to be a more robust phenomenon than that based on negative facial expressions (e.g. anger, sadness, fear [13], [14], [20], [42], [43]; although see [44], [45] for converse results). Also, it is possible, given the near-significant correlation between LSAS scores and participants' ratings of anger-primed versus smile-primed characters (S-A liking), that future experimenters using more strongly angry stimuli, or more socially anxious participants, may find significant priming effects from both smiling and angry-face primes. Nevertheless, the reason for the superiority of smile-primes in generating elevated liking ratings in the present study remains to be established. For example, it is possible that the morphological characteristics of smiling expressions make them more accessible to early stage visual processing mechanisms than most angry expressions. It is also possible that the use of neutral-face primes as “non-affective” comparison primes may be important, as neutral faces can often be interpreted as somewhat threatening or negative [46]. If such an interpretative tendency operates at the very earliest stages of visual processing (i.e. at 10 msec exposures), we could expect to see greater perceived differences between smiling and “neutral” primes, than between angry and neutral ones. And if the negative interpretation of neutral face primes is exaggerated in social anxiety, we would expect to see an elevated priming effect in these individuals, in particular [47], [48].

Even regardless of these possibilities, our finding that neither affective priming overall, nor the association between priming and trait social anxiety, occurred at all in response to angry face primes, does not fit comfortably with evidence from other experimental paradigms that explore pre-attentive processing in anxiety. Based on data from a variety of attention-based methodologies such as the dot-probe and spatial-cueing tasks [49], [50], a major area of agreement across a range of models of attentional biases in anxiety is the potentiation of a pre-attentive threat-detection system in anxious individuals [51]. Social phobia and social anxiety are associated with early-stage enhancement of attention towards threat stimuli [24], [25], [52]. In developing the present investigation of the association between social anxiety and subliminal affective priming, we anticipated that our findings would contribute to an extended understanding of this pre-attentive threat-detection process, by demonstrating whether trait social anxiety is associated with enhancement of valenced affective responses to very briefly presented emotive images. The fact that the responses identified were associated with positive (smiling) but not threat (angry face) images, points to the possibility that the evaluative process associated with subliminal affective priming, and those found more commonly in a range of threat detection paradigms, may be the result of separate early-stage mechanisms. Further investigations will be needed to confirm this, and to demonstrate how these early-stage evaluative processes differ. One possibility is that the smiling face priming effect is generated indirectly as a result of briefly presented images engendering fleeting smile-like expressions in the observer (i.e. as a result of automatic perception-action mimicry), which in turn bias evaluative judgements of temporally contiguous Chinese characters [53]. Socially anxious participants may show elevated priming due to a greater tendency to automatically generate smiles in such situations, or to give biased evaluations as a result of producing these expressions. This possibility of the subliminal affective priming effect being behaviourally mediated could be tested in future studies by preventing participants from smiling (e.g. by requiring them to hold a pen in their lips – [53]) during SAP tasks. A tentative piece of evidence in favour of this hypothesis already exists in the form of a recent study of schizophrenic patients suffering from affective flattening (in which facial and other expressive features of emotions are dampened, even though emotional feelings are not suppressed), which found an absence of smile-based priming in patients, but normal (positive) smile-based priming in healthy controls [43].

Previous investigations of SAP in psychiatric disorders have yielded mixed findings. Two studies found an absence of subliminal affective priming in clinically depressed inpatients, as compared to healthy controls [42], [54], pointing to the possibility that very severe mood disorder may result in a complete shut-down of early-stage affective processing of the affective salience of emotive faces. Research with in-patient schizophrenics, on the other hand, has found partially enhanced SAP effects [43], [55]. For example, [55] found that schizophrenic participants showed elevated priming (relative to control participants) to negative facial expression primes in particular, and the authors suggested that the stronger priming in these patients may be indicative of a heightened sensitivity to negative stimuli. Similarly, in another experiment, anhedonic schizophrenic patients were found to show priming to sad (as compared to neutral) facial expressions, even though all other participant groups showed no priming at all to these stimuli [43]. These same patients also showed incongruent priming to smiling faces (i.e. they rated subsequently presented Chinese characters more negatively than those that followed neutral face primes). The present study is the first to demonstrate an association between degree of subliminal affective priming and emotional traits in a non-clinical population, indicating that even relatively small, self-reported differences in social behaviour and experience can be associated with variation in this very rapid form of valenced, pre-attentive processing. Indeed, the overall SAP effect found here was probably largely dependent on socially anxious individuals within the participant sample; it is possible that the priming effects found in previous studies could also be predominantly attributable to similarly anxious individuals within their respective participant populations [11]–[15]. Whether it is trait social anxiety itself, or state anxiety induced by the testing situation amongst trait socially anxious individuals, which mediates this modulation is not clear. Nevertheless, future investigations of the mechanisms of facial-expression based affective priming may benefit from the use of socially anxious participant groups to magnify this relatively subtle effect. Because of the higher levels of social anxiety in females as compared with males, all female samples may also reveal higher levels of SAP. And finally, future studies may discover even greater modulation of affective priming to facially expressed affect in clinically diagnosed social anxiety disorder.

When the SAP effect was first observed, it was thought that the briefly presented facial expression primes modified participants' liking of Chinese characters by triggering fleeting, diffuse affective feeling states, which were attributed to the characters in the absence of conscious registration of their actual causes (i.e. the primes themselves) [11]. However, a number of subsequent studies have raised doubts that the process has any involvement of subjectively experienced affective feelings at all. For example, [15] found that extended sequences of unseen smiling primes generated SAP effects, but no concomitant changes in self-reported mood were detected. And [12] found that participants were not able to “attribute away” the effects of briefly presented primes when they were made aware that their feeling states might misdirect their Chinese-character evaluations. Our present finding that individual variation in the trait of mood awareness (a tendency to attend to and consider ones own affective states, and the causes of emotions) is not associated with affective priming concurs with Winkielman et al's [27] conclusion that the process of affective priming is not likely to be a conscious one, as we would expect participants with higher levels of mood awareness to be more susceptible to priming effects if they were mediated by subtle feeling states. It also confirms that the modulation of affective priming by social anxiety is not mediated by higher levels of mood awareness in socially anxious individuals. While future studies may be able to make stronger tests of whether the process of affective priming operates on the basis of fleeting changes in consciously experienced affect, the findings of the present study do not offer support for this possibility.

In conclusion, contemporary theories of anxiety emphasise the importance of rapid, pre-attentive evaluations of visual stimuli [56]–[59] as a process which allows anxious individuals to differentially allocate attentional resources to potential threats, and in recent years, modification of this process has become a focus of therapeutic research [60]. The present study aimed to further our understanding of pre-attentive evaluations by investigating whether anxiety is associated with modulations of the subliminal affective priming (SAP) effect, in which pre-attentive processing of facial expressions divert immediately subsequent affective (i.e. valenced) responses to unrelated stimuli. Our results demonstrated that trait social anxiety is linked to the degree of SAP shown to smiling faces in particular. These findings, however, do not readily support the view that the pre-attentive evaluations of threat inferred from other paradigms (such as attentional probe tasks) are also involved in subliminal affective priming. Instead, we suggest that the SAP effect may operate via a separate pre-attentive evaluative process which operates automatically and unconsciously to subtly influence evaluative decisions regarding contiguously presented stimuli.

## References

[1] De Gelder B, Tamietto M, van Boxtel G, Goebel R, Sahraie A (2008). Intact navigation skills after bilateral loss of striate cortex.. Curr Biol.

[2] De Gelder B, Vroomen J, Pourtois G, Weizcrantz L (1999). Non-conscious recognition of affect in the absence of striate cortex.. Neuroreport.

[3] Tamietto M, De Gelder B (2010). Neural bases of the non-conscious perception of emotional signals.. Nat Rev Neurosci.

[4] Pessoa L, Adolphs R (2010). Emotion processing and the amygdala: from low road to many roads of evaluating biological significance.. Nat Rev Neurosci.

[5] Morris JS, Ohman A, Dolan RJ (1999). Conscious and unconscious learning in the human amygdala.. Nature.

[6] Ohman A, Lewis M, Haviland JM (1993). Fear and anxiety as emotional phenomenon: Clinical phenomenology, evolutionary perspectives, and information-processing mechanisms.. Handbook of Emotions.

[7] Esteves F, Dimberg U, Ohman A (1994). Automatically elicited fear: conditioned skin conductance responses to masked facial expressions.. Cog Emo.

[8] Dimberg U, Peterson M (2000). Facial reactions to happy and angry facial expressions: evidence for right hemisphere dominance.. Psychophysiology.

[9] Klauer KC, Musch J (2003). Affective priming with subliminally presented pictures.. Can J Exp Psychol.

[10] Mogg K, Bradley BP (1999). Orienting of attention to threatening facial expressions presented under conditions of restricted awareness.. Cogn Emo.

[11] Murphy ST, Zajonc RB (1993). Affect, cognition and awareness: Affective priming with optimal and suboptimal stimulus exposures.. J Pers Soc Psychol.

[12] Winkielman P, Zajonc RB, Schwarz N (1997). Subliminal affective priming resists attributional interventions.. Cogn Emo.

[13] Rotteveel M, de Groot P, Geutskens A, Phaf RH (2001). Stronger suboptimal than optimal affective priming?. Emotion.

[14] Wong PS, Root JC (2003). Dynamic variations in affective priming.. Consc Cogn.

[15] Winkielman P, Berridge KC, Wilbarger JL (2005). Unconscious affective reactions to masked happy versus angry faces influence consumption behaviour and judgements of value.. Pers Soc Psychol Bull.

[16] Scherer KR, Scherer KR, Schorr A, Johnstone T (2001). Appraisal considered as a process of multilevel sequential checking.. Appraisal Processes in Emotion. Theory, Methods and Research.

[17] Rolls ET (2005). Emotion Explained.

[18] Gilboa-Schetman E, Foa EB, Amir N (1999). Attentional biases for facial expressions in social phobia: The face in the crowd paradigm.. Cogn Emo.

[19] Horley K, Williams LM, Gonzalvez C, Gordon E (2003). Social phobics do not see eye to eye: A visual scanpath study of emotional expression processing.. J Anx Dis.

[20] American Psychiatric Association (1994). Diagnostic and Statistical Manual of Mental Disorders, 4^th^ ed.

[21] Furmark T (2002). Social phobia: Overview of community surveys.. Acta Scand.

[22] Kessler RC, Berglund P, Demler O, Jin R, Merikangas KR (2005). Lifetime prevalence and age of onset distributions of DSM disorders in the National Comorbidity Survey Replication.. Arch Gen Psychiat.

[23] Miskovic V, Schmidt LA (2012). Social fearfulness in the human brain.. Neurosci Biobehav Rev.

[24] Leary MR (1983). A brief version of the fear of negative evaluation scale.. Pers Soc Psychol Bull.

[25] Liebowitz MR (1987). Social phobia.. Mod Prob Pharm.

[26] Dannlowski U, Ohrmann P, Bauer J, Kugel H, Arolt V (2007). Amygdala reactivity predicts automatic negative evaluations for facial emotions.. Psych Res: Neuroim.

[27] Staugaard SR (2010). Threatening faces and social anxiety: A literature review.. Clin Psy Rev.

[28] Machado-de-Sousa JP, Arrais KC, Alves NT, Chagas MHN, de Meneses-Gaya C (2010). Facial affect processing in social anxiety: Tasks and stimuli.. J Neurosci Meth.

[29] Stevens S, Rist F, Gerlach AL (2009). Influence of alcohol on the processing of emotional facial expressions in individuals with social phobia.. Brit J Clin Psychol.

[30] Mogg K, Bradley BP (2002). Selective orienting of attention to masked threat faces in social anxiety.. Beh Res Ther.

[31] Mogg K, Philppot P, Bradley BP (2004). Selective attention to angry faces in clinical social phobia.. J Abnorm Psychol.

[32] Wieser MJ, Pauli P, Alpers GW, Muhlberger A (2009). Is eye to eye contact really threatening and avoided in social anxiety? An eye tracking and psychophysiology study.. J Anx Dis.

[33] Winkielman P, Knutson B, Paulus M, Trujillo JL (2007). Affective influence on judgements and decisions: Moving towards core mechanisms.. Review of General Psychology.

[34] Swinkels A, Giuliano TA (1995). The measurement and conceptualization of mood awareness: Monitoring and lablling one's mood states.. Pers Soc Psychol Bull.

[35] Coffey E, Berenbaum H, Kerns JG (2003). The dimensions of emotional intelligence, alexithymia and mood awareness: Associations with personality and performance on an emotional stroop task.. Cogn Emo.

[36] Schwarz N, Clore GL (1983). Mood, misattribution, and judgements of well-being – informative and directive functions of affective states.. J Pers Soc Psychol.

[37] Anders S, Birbaumer N, Sadowski B, Erb M, Mader I (2004). Parietal somatosensory association cortex mediates affective blindsight.. Nat Neurosci.

[38] Holender D (1986). Semantic activation without conscious identification in dichotic listening, parafoveal vision, and visual masking – a survey and appraisal.. Behav Brain Sci.

[39] Ekman P, Friesen W (1976). Pictures of facial effect.

[40] Schneider W, Eschman A, Zuccolotto A (2000). E-prime Reference Guide.

[41] Heimberg RG, Horner KJ, Juster HR, Safren SA, Brown EJ, Schneier FR (1999). Psychometric properties of the Liebowitz Social Anxiety Scale.. Psy Med.

[42] Dannlowski U, Kersting A, Donges U-S, Lalee-Mantzel J, Arolt V (2006). Masked facial affect priming is associated with therapy response in clinical depression.. Euro Arch Clin Psych Clin Neurosci.

[43] Suslow T, Roestel C, Arolt V (2003). Affective priming in schizophrenia with and without affective negative symptoms.. Euro Arch Psychiat Clin Neurosci.

[44] Murphy ST, Monahan JL, Zajonc RB (1995). Additivity of nonconscious affect:Combined effects of priming and exposure.. J Pers Soc Psychol.

[45] Sato W, Aoki S (2006). Right hemispheric dominance in processing of unconscious negative emotion.. Brain and Cognition.

[46] Lee E, Kang JI, Park IH, Kim JJ, An SK (2008). Is a neutral face really evaluated as being emotionally neutral?. Psychiat Res.

[47] Yoon K, Zinbarg RE (2008). Interpreting faces as threatening is a default mode for socially anxious individuals.. J Abnorm Psychol.

[48] Joorman J, Gotlib IH (2006). Is this happiness I see? Biases in the identification of emotional facial expression in depression and social phobia.. J Abnorm Psychol.

[49] MacLeod C, Mathews A, Tata P (1986). Attentional bias in emotional disorders.. J Abnorm Psychol.

[50] Fox E, Russo R, Bowles R, Dutton K (2001). Do threatening stimuli draw or hold visual attention in subclinical anxiety?. J Exp Psychol: Gen.

[51] Cisler JM, Koster EHW (2010). Mechanisms of attentional biases towards threat in anxiety disorders: An integrative review.. Clin Psychol Rev.

[52] Garner M, Mogg K, Bradley BP (2006). Orienting and maintenance of gaze to facial expressions in social anxiety.. J Abnorm Psychol.

[53] Strack F, Martin LL, Stepper S (1988). Inhibiting and facilitating conditions of the human smile: a non-obtrusive test of the facial feedback hypothesis.. J Pers Soc Psychol.

[54] Koschack J, Hoschel K, Irle E (2003). Differential impairments of facial affect priming in subjects with acute or partially remitted major depressive episodes.. J Nerv Ment Dis.

[55] Hoschel K, Irle E (2001). Emotional priming of facial affect identification in schizophrenia.. Schiz Bull.

[56] Williams JMG, Watts FN, MacLeod C, Matthews A (1988). Cognitive psychology and emotional disorders.

[57] Williams JMG, Watts FN, MacLeod C, Matthews A (1997). Cognitive psychology and emotional disorders (2^nd^ Edition).

[58] Mogg K, Bradley BP (1998). A cognitive-motivational analysis of anxiety.. Beh Res Ther.

[59] Bar-Haim Y, Lamy D, Pergamin L, Bakermans-Kranenburg MJ, van IJzendoorn MH (2007). Threat-related attentional bias in anxious and non-anxious individuals: A meta-analytic study.. Psych Bull.

[60] Mathews A, MacLeod C (2002). Induced processing biases have causal effects on anxiety.. Cogn Emo.

